# Progranulin (PGRN) as a regulator of inflammation and a critical factor in the immunopathogenesis of cardiovascular diseases

**DOI:** 10.1186/s12950-023-00327-0

**Published:** 2023-01-19

**Authors:** Ali Saeedi-Boroujeni, Daryush Purrahman, Ali Shojaeian, Łukasz A. Poniatowski, Fatemeh Rafiee, Mohammad-Reza Mahmoudian-Sani

**Affiliations:** 1 Department of Microbiology, School of Medicine, Abadan University of Medical Sciences, Abadan, Iran; 2 Thalassemia and Hemoglobinopathy Research Center, Health Research Institute, Ahvaz Jundishapur University of Medical Sciences, Ahvaz, Iran; 3 Research Center for Molecular Medicine, Hamadan University of Medical Sciences, Hamadan, Iran; 4 Department of Neurosurgery, Dietrich-Bonhoeffer-Klinikum, Neubrandenburg, Germany; 5 Zanjan Metabolic Diseases Research Center, Zanjan University of Medical Science, Zanjan, Iran; 6 Clinical Research Development Unit, Golestan Hospital, Ahvaz Jundishapur University of Medical Sciences, Ahvaz, Iran

**Keywords:** Progranulin, Inflammation, Immunopathogenesis, Cardiovascular diseases, Therapy

## Abstract

Immune dysregulation has been identified as a critical cause of the most common types of cardiovascular diseases (CVDs). Notably, the innate and adaptive immune responses under physiological conditions are typically regulated with high sensitivity to avoid the exacerbation of inflammation, but any dysregulation can probably be associated with CVDs. In this respect, progranulin (PGRN) serves as one of the main components of the regulation of inflammatory processes, which significantly contributes to the immunopathogenesis of such disorders. PGRN has been introduced among the secreted growth factors as one related to wound healing, inflammation, and human embryonic development, as well as a wide variety of autoimmune diseases. The relationship between the serum PGRN and TNF-α ratio with the spontaneous bacterial peritonitis constitute one of the independent predictors of these conditions. The full-length PGRN can thus effectively reduce the calcification of valve interstitial cells, and the granulin precursor (GRN), among the degradation products of PGRN, can be beneficial. Moreover, it was observed that, PGRN protects the heart against ischemia-reperfusion injury. Above all, PGRN also provides protection in the initial phase following myocardial ischemia-reperfusion injury. The protective impact of PGRN on this may be associated with the early activation of the PI3K/Akt signaling pathway. PGRN also acts as a protective factor in hyperhomocysteinemia, probably by down-regulating the wingless-related integration site Wnt/β-catenin signaling pathway. Many studies have further demonstrated that SARS-CoV-2 (COVID-19) has dramatically increased the risks of CVDs due to inflammation, so PGRN has drawn much more attention among scholars. Lysosomes play a pivotal role in the inflammation process, and PGRN is one of the key regulators in their functioning, which contributes to the immunomodulatory mechanism in the pathogenesis of CVDs. Therefore, investigation of PGRN actions can help find new prospects in the treatment of CVDs. This review aims to summarize the role of PGRN in the immunopathogenesis of CVD, with an emphasis on its treatment.

## Cardioimmunology

**Fig. 1:**
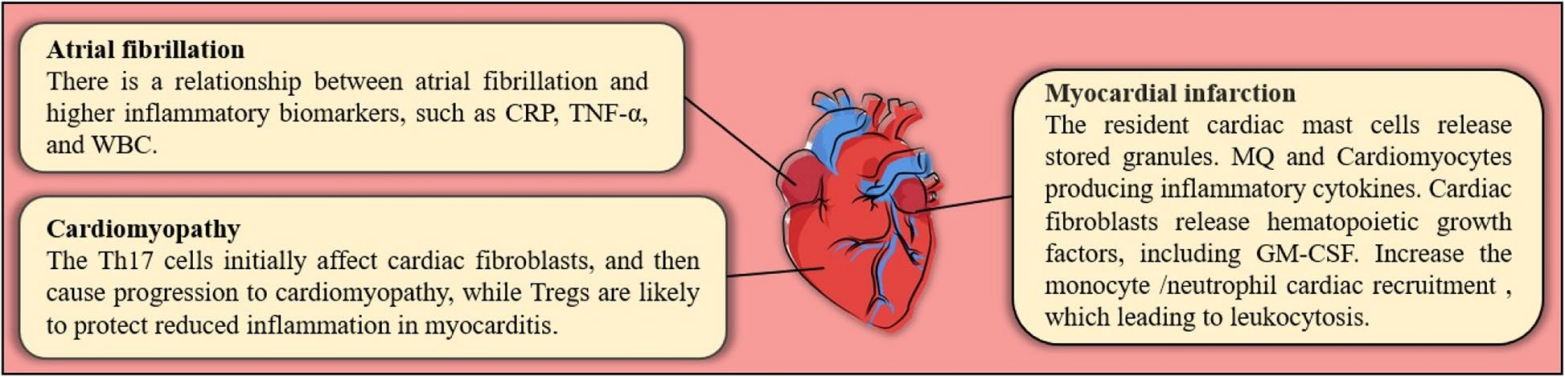
Summary diagram of immunopathogenesis involved in cardiovascular disease

Many types of cardiovascular diseases (CVDs) are associated with complex immune responses that can significantly contribute to their progression and remission. In recent years, extensive research has established that some immune cells either reside in the heart or have very complicated interactions with cardiomyocytes through permanent blood circulation. Studies have further confirmed the presence of mast cells, T and B lymphocytes, and neutrophils in the heart. Macrophages and dendritic cells are also present in normal heart valves. Besides, the pericardium contains various leukocytes, including macrophages and B cells [1]. The heart of a healthy adult rat accordingly holds all major classes of leukocytes, e.g., mononuclear phagocytes, neutrophils, and T and B lymphocytes [2]. Therefore, immune cells are typically expected to play a vital role in regulating the immune system responses in the heart. In all types of CVDs, inflammation has been further documented as one of the critical events in the initiation and spread of the pathological processes. For example, shortly after the onset of ischemia, the resident cardiac mast cells release the stored contents of their granules [3], whereas macrophages and cardiomyocytes embark on the production of inflammatory cytokines. This covers the production of several cytokines and chemokines including interleukin 1 (IL-1), interleukin 6 (IL-6), tumor necrosis factor- alpha (TNF-α) and chemokine (C-C motif) ligand 2 (CCL2) [4]. Cardiac fibroblasts also release hematopoietic growth factors, including granulocyte-macrophage colony-stimulating factor (GM-CSF) [5]. These events accordingly increase monocyte and neutrophil recruitment, leading to leukocytosis, which constitutes one of the independent risk factors for CVDs [6]. In myocarditis, T cell differentiation can crucially contribute to aggravating or alleviating such conditions. Many studies have thus demonstrated that the T helper 17 (Th17) cells initially affect cardiac fibroblasts and cause the progression of cardiomyopathy, while regulatory T cells (Tregs) are likely to protect and reduce inflammation in myocarditis (Fig. 1) [7, 8]. Interestingly, atrial fibrillation (AF) occurrence elevates under inflammatory conditions, such as sepsis and rheumatoid arthritis [9]. Based on previous research, there is a relationship between AF and higher inflammatory biomarkers, such as C-reactive protein (CRP), TNF-α and white blood count [10]. The endothelial cells in the blood vessels of the heart produce adhesion molecules, such as vascular cell adhesion molecule 1 (VCAM-1) and selectins, soon after myocardial infarction (MI) within minutes. In this case mast cells release TNF-α, and molecular damage-associated molecular patterns (DAMPs), such as high mobility group box 1 (HMGB1), adenosine triphosphate (ATP), calprotectin (S100A8/A9) whereas other cells, including macrophages and fibroblasts, consequently produce cytokines and chemokines, such as CCL2 and IL-1. These events then lead to a massive influx of neutrophils and monocytes from the circulation into the heart tissue in 1 day. In 3-7 days, neutrophils withdraw from the heart tissue. Monocytes also accumulate, produce transforming growth factor beta (TGFβ), interleukin 10 (IL-10) and vascular endothelial growth factor (VEGF) as well as give rise to collagen production by fibroblasts, reduced inflammation via Tregs, and neoangiogenesis. Such events have been thus far reported in other CVDs, including myocarditis. SARS-CoV-2 (COVID-19) is an infection with an inflammatory component, where aggravation and the cytokine storm phenomenon lead to multiple organ failures. The inflammatory issues related to the COVID-19 infection also raised the role of inflammation in the pathogenesis of CVDs. This review article accordingly aims to investigate the role of PGRN in the regulation of the immune responses, especially the inflammatory processes, in various types of CVDs. In order to present a comprehensive overview of the problem, we will perform a thorough analysis of the structure and functions of the PGRN and present its role in the physiological processes related to CVDs with particular attention paid to each stage of the pathology and its subsequent consequences, and also possible clinical implications.

## Progranulin (PGRN)

PGRN constitute the pleiotropic growth factor and a significant anti-inflammatory molecule with an important role in maintaining and regulating homeostatic dynamics in normal tissue development, regeneration, host defense response and proliferation [11]. Expression of PGRN is observed in the immune cells, epithelial cells, chondrocytes and neurons [12, 13]. PGRN can thus directly bind to TNF receptors (TNFRs) and disrupt the interactions of TNF-α with such receptors [14–16]. The increased serum PGRN levels were observed in different diseases, which are characterized by the presence of chronic low-grade inflammation (i.e., meta-inflammation), like atherosclerosis [17], neurodegenerative diseases [18], breast cancer [19], diabetes mellitus [20], as well as metabolic syndrome [21]. In addition, PGRN has been documented as one of the autocrine growth factor consisting of seven-and-a-half tandem repetitions of the granulin module arranged in the P-G-F-B-A-C-D-E sequence [22]. The proteolytic degradation of the PGRN holoprotein takes place both in the intra- and extracellular spaces, leading to the release of individual 45-granulin precursor (GRN) fragments contained in the 56-57 amino acid residues with a molecular weight of about ~ 6 kDa, which occur in both individual and combined forms (~ 6-25 kDa) after proteolysis [23, 24]. Proteolytic degradation is also mediated by various intra- and extra-cellular serine and threonine proteinases, such as matrix metalloproteinase 9 (MMP-9), matrix metalloproteinase 12 (MMP-12), matrix metalloproteinase 14 (MMP-14), disintegrin, and metalloproteinase with the thrombospondin 7 motif (ADAMTS-7), neutrophil elastase (ELANE), and proteinase 3 (PRTN3), wherein the mutual interactions are regulated by feedback loops [25, 26]. PGRN is a critical regulator in various biological processes, like wound healing [27] and bone regeneration [28]. The PGRN knockout (KO) mice had shown the exacerbation of inflammatory diseases, including atherosclerosis and rheumatoid arthritis [16]. Considering the stage and the factors contributing to the tissue microenvironment, researchers have indicated the pro- or anti-inflammatory activities of PGRN, which may be protective or harmful for humans [29]. Administering the recombinant human PGRN (rPGRN) had thus largely reduced the inflammatory response in the cardiovascular system of animals with rheumatoid arthritis. On the other hand, deficiency in the PGRN level had worsened atherosclerosis in apolipoprotein E (ApoE) KO mice [16].

## TNF-α response regulation by PGRN in cardiovascular diseases

The reduction in the PGRN/TNF-α ratio was observed in the course of many inflammatory diseases. In this case, the PGRN/TNF-α ratio has been shown to be one of the independent predictors of high systolic blood pressure (SBP), implying the importance of the inflammatory components involved in hypertension. TNF-α acts as one of the crucial inflammatory cytokines imparting a significant contribution to the start and continuance of immunological reaction. Extensive research has further demonstrated the higher level of TNF-α in CVDs, as well as in conditions associated with low-grade inflammation, such as obesity [30], diabetes mellitus [31], metabolic syndrome [32], and atherosclerosis [33]. Hypertension constitutes an independent risk factor for cardiovascular, brain, and kidney diseases. For more than 50 years, accumulating literature has further emphasized the contribution of inflammation to hypertension pathogenesis. The immune cells are similarly found in the veins and kidneys of people with hypertension. In this case, vasculitis can centrally contribute to the development of essential hypertension [34]. Furthermore, low-grade inflammation plays a leading role in the pathogenesis of isolated systolic and systolic-diastolic hypertension. The anti-inflammatory activities of PGRN can be thus attributed to the inhibition of the mitogen-activated protein kinase (MAPK) signaling and TNFR-mediated nuclear factor-κB (NF-κB) as well as competitive binding to TNF-R2, which is observed in the bone marrow-derived macrophages [22]. A significant positive correlation had been accordingly observed between the serum PGRN level and both SBP and diastolic blood pressure (DBP) in the diabetic patients with microangiopathy [35]. Accordingly, PGRN, as one of the competitive molecules of TNF-α, is expressed in case of hypertension secondary to higher inflammatory cytokines, especially TNF-α. Moreover, the serum PGRN/TNF-α ratio has been shown to be crucial for exploring the inflammatory microenvironment in patients. The lower ratio of PGRN/TNF-α in the cases with hypertension, compared with healthy samples, accordingly suggests the regulation and control of TNF-α through PGRN [36].

## Role of PGRN in the immunopathogenesis of calcific aortic valve disease

**Fig. 2:**
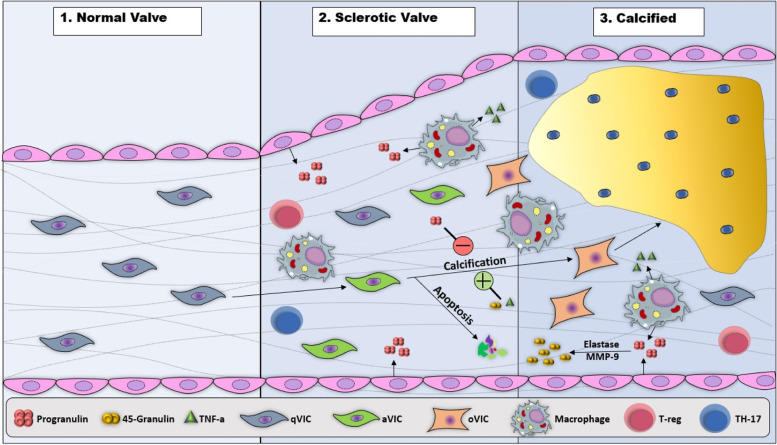
The schematic representation of the histological change progression during calcific aortic valve disease, wherein PGRN can suppress calcification and disease progression whereas GRN and TNF-α could promote it

Calcific aortic valve disease (CAVD) constitute common condition which affect 25% of people aged over 65. CAVD and atherosclerosis share common risk factors. Despite the common causes of both disorders (e.g., smoking, hypertension, dyslipidemia, diabetes mellitus, metabolic syndrome, and inflammation), the molecular mechanisms associated with CAVD are still not well recognized, so no effective drug treatment has been found for this condition, except valve replacement [37, 38]. The PGRN expression was further observed in the aortic valve and its level is largely increased in patients with CAVD. In addition, PGRN vitally contributes to chondrocyte proliferation, ectopic calcification, and differentiation [12, 39, 40]. The microarray results confirmed that PGRN was one of the CAVD-associated molecules. However, the PGRN contribution to CAVD progression is still not fully understood. Valve interstitial cells (VIC) constitute a fibroblastic population, but their differentiation has been observed into a myofibroblastic phenotype under cell culture. Such cells are fundamental in the calcification process, and could be essential for the mechanisms involved in heart valve calcification. The human VICs and the primary porcine ones isolated from human/porcine and genetically modified mice had been exploited for investigating the PGRN contribution to CAVD development as well as discovering the related molecular mechanisms. The study results had indicated that the full-length PGRN had effectively reduced the GRN-mediated calcification. The PGRN degradation products and VICs had consequently accelerated the calcification of VICs [41]. PGRN/GRN are thus among the new factors in the pathogenesis of VICs and constitute a good therapeutic target to treat and prevent CAVD. In addition, the PGRN expression has been observed in VICs that increase in the valves of the patients with CAVD [42, 43]. The GRN has largely enhanced the expression of the calcified valve. Using an in vitro model, valve calcification had implied the higher levels of the GRN expression, while the full-length PGRN had dwindled. The study findings correspondingly revealed that the full-length PGRN and GRN had been involved in the CAVD pathogenesis [44, 45]. PGRN also prevents the aortic valve calcification in CAVD through the inhibition of the osteogenic differentiation of VICs and myofibroblastic transition [41]. In this line, one investigation found that PGRN could accelerate the regeneration of bones by inhibiting the inflammatory response and accelerating the osteogenic differentiation [46]. CAVD may thus have a specific inflammatory response to calcification. Furthermore, TNF-α enhances the osteogenic differentiation of VICs in CAVD (Fig. 2). On the contrary, TNF-α moderates the osteogenic differentiation capacity in bone remodeling, and such a difference can be prompted by the special structural characteristics of the valve [47, 48].

## Regulation of Wnt/β-catenin signaling pathway by PGRN

PGRN down-regulates the Wnt/β-catenin signaling pathway, which is evolutionarily conserved developmental signaling cascade with a robust performance in the regulation of various biological processes for tissue development and human disease pathogenesis [49]. Even though the Wnt/β-catenin signaling pathway in the kidney has been shown to be essential for nephron formation, and is functionally silenced after differentiation in the adult kidneys, new documents suggest that the reactivation of the Wnt/β-catenin signaling after renal injuries is of great importance in their acceleration. The activity of this pathway also affects regulatory molecules that covers transient receptor potential channel 6 (TRPC6), angiotensin II receptor type 1 (AT1) and zinc finger protein SNAI1. Moreover, Wnt and β-catenin is associated with the activation of the podocytes of patients with diabetic nephropathy and focal segmental glomerulosclerosis, indicating the clinical association of this pathway with the human proteinuric renal diseases [50]. The Wnt/β-catenin signaling pathway in the cardiovascular system is further implicated in myocardial remodeling following pathological damage [51]. In this respect, Nakagawa et al. [52] demonstrated that the continuous activation of the Wnt/β-catenin signaling pathway in the endothelial cells was possibly the reason for heart failure. While functional genomic analyses have highlighted the role of the Wnt/β-catenin signaling pathway in the PGRN deficiency in the human fetal neural progenitors [53]. PGRN can down-regulate the Wnt1/β-catenin signaling pathways in hyperhomocysteinemia, with various activities in diverse cell types through processes such as the regulation of the cell fate determination, the expression of the podocyte differentiation markers, as well as the permeability of the endothelial cells. PGRN is also assumed as a protective factor in hyperhomocysteinemia, possibly by negatively regulating the Wnt/β-catenin signaling pathway [54].

## Therapeutic potential of PGRN in hyperhomocysteinemia

**Fig. 3:**
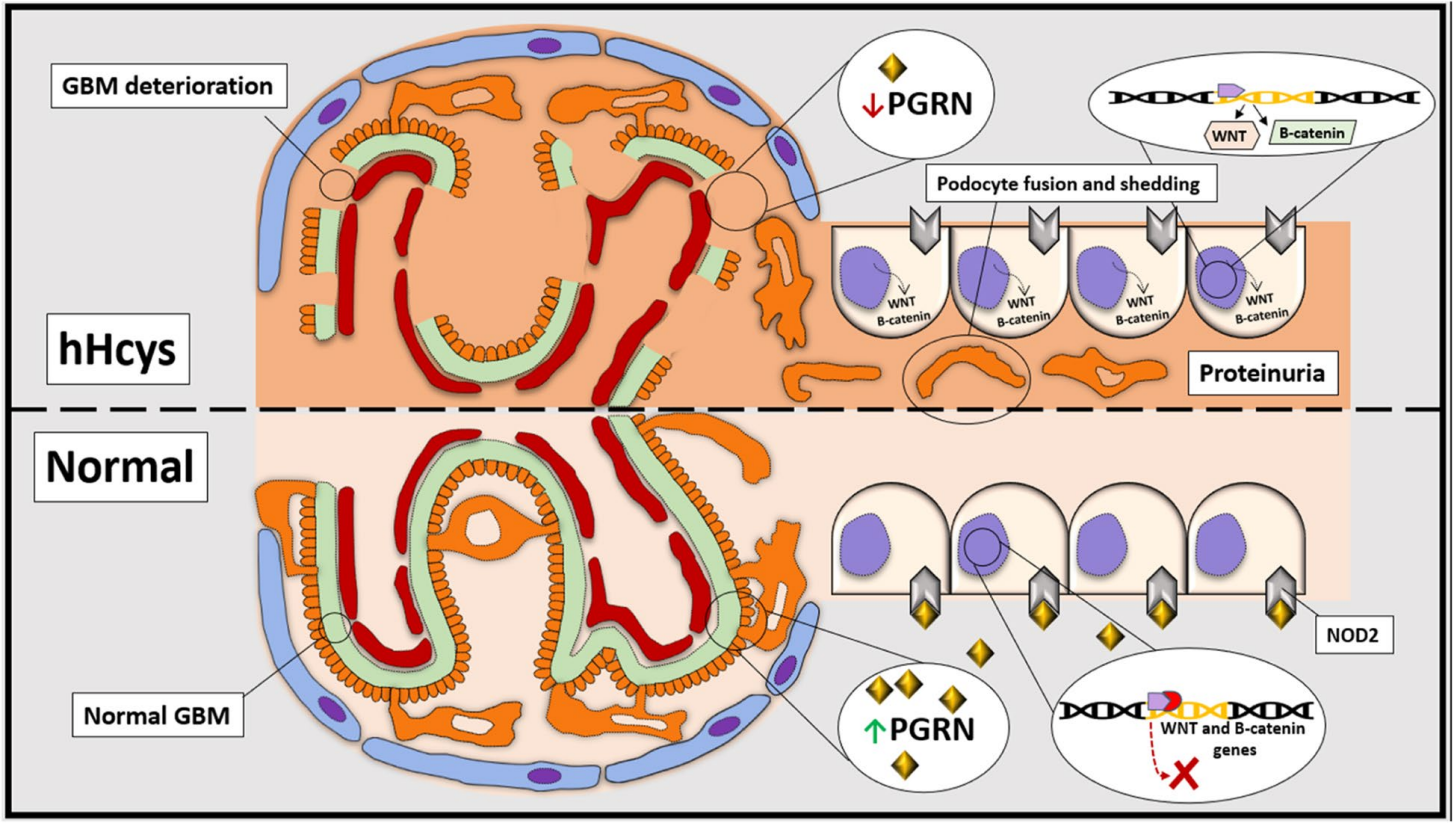
Comparison between normal conditions and hyperhomocysteinemia in the proximal tube and bowman capsule with focus on the role of PGRN. The absence of PGRN In hyperhomocysteinemia activates the Wnt/β-catenin signaling pathway, which play a significant role in this pathological condition. However, presence of PGRN under normal conditions suppresses the Wnt/β-catenin expression

Homocysteine is a sulfhydryl-containing amino acid that is not obtained from diet, but synthesized as an intermediate metabolite in the methionine cycle. As well, hyperhomocysteinemia in the absence of kidney disease indicates a disorder in sulfur amino acid metabolism, which occurs as a result of a deficiency in vitamins (folate, B12 and B6) or genetic defects. Besides, hyperhomocysteinemia is associated with inflammation and atherosclerosis. Based on epidemiological and clinical research, hyperhomocysteinemia has been introduced as one of the independent leading risk factors for developing CVDs and end-stage renal disease [55, 56]. Even though researchers have so far applied several methods to reduce the hyperhomocysteinemia levels in clinical trials and experimental studies, there are no efficient treatments for the complete prevention of renal injury and hyperhomocysteinemia-induced cardiac conditions [57, 58]. PGRN acts as one of the down-regulators of in acute kidney injury [59]. Nonetheless, the contribution of PGRN on the pathogenesis of hyperhomocysteinemia is unknown. Significant reduction in the PGRN levels had been observed in the heart and kidney in the rodent model of hyperhomocysteinemia where podocytes essentially contribute to the circulation of the glomerular basement membrane, the regulation of the glomerular filtration, as well as the maintenance of the glomerular filtration barrier [60, 61]. The damage to the above-mentioned components leads to the permeability of the glomerular capillary, resulting in glomerular disease and proteinuria. PGRN deficiency exacerbates the podocyte fusion, podocyte shedding, inflammatory response, glomerular basement membrane destruction and higher proteinuria in hyperhomocysteinemia (Fig. 3) [54]. Additionally, conducted research has established a relationship between mortality rate and cardiovascular complications caused by hyperhomocysteinemia [62]. According to animal research, hyperhomocysteinemia is involved in cardiac hypertrophy [63]. The PGRN deficiency also exacerbates the hyperhomocysteinemia-induced left ventricular dilation and hypertrophy in a rodent models, suggesting that PGRN is one of the target molecules vital to maintain cardiovascular function [54].

## Immunoregulatory effects of PGRN in myocardial infarction

Worldwide, ischemic heart disease is the main cause of mortality in humans [64, 65]. According to previous studies, growth factors or adipokines are secreted by adipose tissues play significant role in heart function [66, 67]. Multiple adipokines exhibit cardioprotective features, but some of them have been linked to the pathogenesis of CVD [67]. Adipokines, consisting of adiponectin and some C1q/TNF-related proteins (CTRPs), accordingly adopt an appropriate function in the heart and blood vessels [67, 68]. Moreover, adipokines protect against myocardial ischemia-reperfusion injury and atherosclerosis via the anti-inflammatory, anti-apoptotic, anti-thrombotic and anti-oxidant effects [67, 69, 70]. In addition, CTRPs apply protective impacts against cardiac remodeling and myocardial ischemia-reperfusion injury through the anti-apoptotic mechanism of action [71, 72]. Researchers have also demonstrated the anti-apoptotic effects of cardioprotective adipokines by activating the PI3K/Akt signaling pathway [71, 72]. As stated, downstream through PI3K/Akt signaling pathway have been shown to be key cell proliferation and survival regulators, involved in prevention of cell apoptosis [73, 74]. In the cardiovascular system, the high expression of PGRN has been also observed in the macrophages in the atherosclerotic arteries and the vascular smooth muscle cells [75]. PGRN plays a role in atherosclerosis, as the major cause of infarction and myocardial ischemia [76]. Nonetheless, its impact on acute myocardial ischemia-reperfusion injury is unknown. In a mouse model of acute myocardial ischemia-reperfusion injury, the PGRN administration had improved cardiac function, reduced inflammation, and protected heart from damage through anti-apoptotic effect. The elevated PGRN expression after myocardial ischemia-reperfusion injury in heart tissue is observed [77]. Using the PI3K/Akt inhibitor, the LY294002 had further revealed that PGRN could exert its cardioprotective impacts via the activation of the PI3K/Akt signaling pathway. However, adipokines in atherosclerosis could suppress atherogenesis, with a protective or preventive role in the coronary heart disease [66]. The PGRN deletion in mice with atherosclerosis was also linked to the higher expression of adhesion molecules and inflammatory cytokines. Furthermore, PGRN had exhibit an anti-atherogenic impact in these mice [16]. In addition, PGRN increases the protection of the vascular endothelium via enhancing nitric oxide by activating the Akt/endothelial nitric oxide synthase (eNOS) signaling pathway [78]. PGRN secreted from the macrophages also generates a complex with apolipoprotein A-I (ApoA-I), contributing to the atherosclerotic plaque stabilization [43]. The PGRN deficiency alters the configuration of high-density lipoprotein (HDL). PGRN also declines the platelet-activating factor, acetylhydrolase, which may reduce the growth of the macrophage foam cells and suppress atherogenesis [43, 79]. Multiple cardioprotective adipokines, like CTRP9 and CTRP3, have also applied the same impacts in the earlier reports [69, 71, 72, 80]. The PGRN effects accordingly diminisheos edema, acute inflammation, congestion, as well as vasodilation in the heart tissue after acute myocardial ischemia-reperfusion injury [77]. During ischemia-reperfusion injury in the brain, PGRN reduces inflammation through the TNF-α-mediated inhibition of the expression of cell adhesion molecules and neutrophil infiltration [15]. Moreover, the reduction of the hypoxia-induced inflammation by PGRN has been observed in the kidney tissue [59]. PGRN probably suppresses inflammation in the acute myocardial ischemia-reperfusion injury in this same way, however, more research is required for detecting its mechanisms. Further studies indicated that the mRNA for PGRN expression in the rat myocardium has strongly increased when myocardial ischemia-reperfusion injury have occurred [77]. The cardioprotective effect of PGRN under in vivo conditions has not yet been fully identified. Cardiomyocyte death due to myocardial ischemia-reperfusion injury is mainly associated with apoptosis [81]. In addition, cells overexpressing B-cell lymphoma 2 (Bcl-2) can largely decline apoptosis and the size of the myocardial infarct once it occurs [82]. Furthermore, the PGRN administration prior to the ischemia induction has enhanced the Bcl-2 expression, suggesting that this anti-apoptotic molecules can act as cardioprotective. Adiponectin was further observed to exhibit similar anti-apoptotic characteristics [72]. Although PGRN increases the Bcl-2 expression, it does not largely change the amount of apoptotic protein p53 [83]. In addition, this protein would protect against oxidative stress in the brain [15, 84] and block NF-κB, as an oxidative stress-responsive transcription factor, resulting in decreased reactive oxygen species (ROS) formation by neutrophils [15]. Hence, the protective effect of PGRN in myocardial ischemia-reperfusion injury may be partly attributed to its antioxidant potentials. Based on the previous reports, the PI3K/Akt signaling pathway protects against M-IRIs [85, 86]. Administration of the recombinant PGRN can further reduce the size of infarct and lower infiltrated neutrophils count after the permanent occlusion of the left coronary artery in rats and enhance cardiac fibrosis and dysfunction after myocardial ischemia-reperfusion injury in rabbits [87]. The PGRN expression is largely marked in the ischemic area of the myocardium, especially the border area following the permanent occlusion of coronary artery. Moreover, cardiomyocyte death in myocardial infarction causes the release of DAMPs, and thus augments inflammatory components and leukocyte migration that accelerate phagocytosis via  removing the matrix debris and the dead cells [88, 89]. The PGRN expression is significantly elevated 24 h after focal cerebral ischemia [90]. The PGRN deficiency also exacerbates tissue injury via the increased infiltration of macrophages and neutrophils after renal ischemia in a mouse model [59]. Therefore, the up-regulated PGRN may be associated with cardioprotection via the regulation of the post-ischemic inflammation. The PGRN expression in the immune cells, like macrophages and neutrophils, is further up-regulated in the process of wound healing under the ischemic condition [27, 90].

## PGRN secretion from neutrophils to regulate myocardial ischemia inflammation

PGRN was observed to merge with the neutrophil marker, NIMP-R14, when the permanent occlusion of the coronary artery is induced, indicating that neutrophils may be a cell-expressing PGRN after this condition. Moreover, the infiltration of the infarct area by neutrophils has been seen in the first few hours following the onset of myocardial ischemia. They generate granule components, like myeloperoxidase and ROS, and then exacerbate tissue damage [91]. Put differently, neutrophil infiltration is needed for resolving the post-myocardial ischemia-reperfusion injury inflammation [92]. Reportedly, neutrophils are associated with post-myocardial ischemia-reperfusion injury inflammation and tissue repair. PGRN thus suppresses neutrophil migration [15]. Therefore, PGRN secreted from neutrophils may regulate post-myocardial ischemia-reperfusion injury inflammation by an autocrine mechanism, and then influence other immune cells. In addition, intravenous administration of the recombinant PGRN largely reduces the size of the infarct 24 h after the permanent occlusion of the left main descending coronary artery (LCA) in mice. The recombinant PGRN significantly suppresses infiltrating neutrophils in the infarcted area 1 day after the permanent occlusion of the LCA. The recombinant PGRN also significantly reduces neuronal injury after focal cerebral ischemia by inhibiting neutrophils [15], and attenuates neutrophil infiltration when renal ischemia occurs [59]. Based on previous research findings, the extra accumulation of neutrophils aggravates the size of the myocardial infarct 24 h after the permanent occlusion of the LCA [93]. Furthermore, reduced neutrophil recruitment decreases the size of the infarct after the permanent occlusion of the LCA and following myocardial ischemia-reperfusion injury [91, 94]. The creation of cardiac fibrosis also results in maintaining tissue integrity in the post-myocardial ischemia-reperfusion injury reparative response [88].

## PGRN attenuation of myocardial fibrosis by activation PI3K/Akt and Wnt/β-catenin inhibition

Fibrosis-induced tissue sclerosis is associated with impaired cardiac contractility and worsening fibrosis in the myocardium, which are associated with post-ischemic cardiac dysfunction [95–97]. The serum PGRN levels are thus associated with the liver fibrosis in cases suffering from non-alcoholic fatty liver disease [98]. PGRN can thus reduce liver fibrosis following chronic injuries to the liver in mice via modulating inflammation [99]. PGRN is also known to be related to tissue fibrosis under inflammatory conditions. Administration of the recombinant PGRN accordingly improves cardiac dysfunction and left ventricular remodeling after myocardial ischemia-reperfusion injury in rabbits. Furthermore, the extent of fibrosis in the myocardium is significantly reduced by the administration of the recombinant PGRN [100, 101]. The administration of recombinant human PGRN largely enhances cardiac function after ischemia by activating the PI3K/Akt signaling pathway [77]. Therefore, the protective impact of PGRN on myocardial ischemia-reperfusion injury might be related to the early activation of PI3K/Akt. The Wnt/β-catenin signaling also significantly contributes to the formation of the post-ischemic fibrosis and cardiac function [102, 103]. Moreover, the alteration of the Wnt/β-catenin signaling speeds up progression of cardiac injury and adverse cardiac remodeling when ischemia occurs [104, 105]. Inhibiting the Wnt/β-catenin signaling thus suppressed cardiac remodeling and fibrosis once ischemia had occurred in the mice [105]. Put differently, PGRN reportedly regulates the Wnt/β-catenin signaling [106]. Hence, the administration of the recombinant PGRN may prevent the worsening of cardiac fibrosis and dysfunction by the Wnt/β-catenin signaling inhibition after myocardial ischemia [107, 108].

## PGRN deficiency and acceleration of age-associated cardiac abnormality

It is widely known that cardiac aging is a complicated pathophysiological process associated with diverse biological changes, like left ventricular hypertrophy, an altered diastolic pattern, as well as diastolic dysfunction and heart rhythm [109, 110]. Likewise, the initiation of ventricular hypertrophy from the cell surface to the organs has been shown to be one of the signs of cardiac aging associated with the accumulation of lipofuscin (age pigment) as well as cardiomyocyte hypertrophy in the human and animal myocardium [111, 112]. Nonetheless, the mechanisms of the relationship between cardiac aging, cardiac dysfunction and hypertrophy have not yet been fully understood [113]. Moreover, the relationship between a heterozygous mutation in PGRN and frontotemporal dementia (FTD) was observed. In this case, PGRN deficiency accelerates brain aging, with the characteristics of astrogliosis, tissue vacuolization, and microgliosis with lipofuscin accumulation [114, 115]. In addition, PGRN deficiency is likely to result in increased age-associated cardiac phenotypes, like cardiac dysfunction and hypertrophy. Even though researchers have so far introduced different mechanisms of age-associated cardiac hypertrophy, β-catenin and the respective downstream genes have been implicated in regulating pathophysiological cardiac hypertrophy in adults [116, 117]. Activating the Wnt/β-catenin signaling pathway accordingly seems to be caused by the binding of one or more serum factors, the frizzled (Fzd) family, to the cell surface receptors. Furthermore, the complement component 1q (C1q) is among the Fzd-binding proteins. Researchers have also observed the increased expression of C1q and serum concentration in various tissues with age. In other words, the C1q binding to Fzd creates the complex, which cleaves the ectodomain of the low-density lipoprotein (LDL) receptor-related protein [113]. Such a cleavage activates and translocates cytosolic β-catenin to the nucleus [118]. Additionally, C1q is enhanced in the microglia and the serum of the PGRN KO mice, which is associated with a neurodegenerative phenotype in the aged mice [119]. The PGRN deficiency further accelerates age-associated cardiac aging, cardiac dysfunction, and hypertrophy, probably through the C1q-induced β-catenin, which increases and is associated with the age-related cardiac abnormality. The PGRN KO mouse hearts also exhibit greater lipofuscin accumulation [120]. PGRN is closely related to the other types of dementia, like Alzheimer’s disease [114, 115, 121]. Moreover, the PGRN deficiency accelerates the age-associated phenotype observed in neurons [122]. The loss of PGRN additionally accelerates intervertebral disc degeneration in aged mice [123]. Increasing age has been further proposed to be one of the main risk factors for cardiac hypertrophy [109, 124, 125]. The PGRN KO mice have exhibited age-dependent cardiac hypertrophy and faster aging in comparison to the wild-type ones [122, 126]. As well, abnormal lipofuscin accumulation has been observed in the heart of aged PGRN KO mice. Despite being short-living cells, some postmitotic cells, like neurons, cardiomyocytes, and skeletal muscle cells, fail to degrade intracellular lipofuscin, which leads to its mass accumulation [127]. In fact, lipofuscin can be detected in some neurons in the brains of young children, though it gradually increases with age [128]. Even though the content of lipofuscin increases with the rise in the weight of the heart [129, 130], there is no information on the association between lipofuscin accumulation and cardiac hypertrophy. C1q also binds to the Wnt receptor Fzd, which cleaves the LDL receptor-related protein 6 (LRP6) and promotes age-associated phenotypes in the skeletal muscle [118]. The age-associated activation of β-catenin accordingly shows the C1q binding to the extracellular cysteine-rich domain of Fzd. The higher activation of β-catenin in the PGRN-deleted cardiomyocytes is further related to the C1q induction. The exogenous PGRN administration also inhibits the C1q binding to Fzd-1 as well as the subsequent β-catenin activation dose-dependently [120]. Hence, PGRN is critical to preventing serum C1q from binding to Fzd-1 in the target cells, so further research must be done to clarify if PGRN directly competes with C1q for binding to Fzd-1. Furthermore, aged PGRN KO mice had shown age-associated cardiac phenotypes, like fibrosis and hypertrophy, which had reduced fraction shortening [120]. The transverse aortic constriction (TAC)-operated PGRN KO mice also have a greater rate of mortality with hypertrophy and exacerbated cardiac dysfunctions. On the other hand, the cardiac aging phenotype had not largely differed between PGRN KO mice and 6-month old wild-type ones, but there was further distinction in the PGRN KO mice than the wild-type ones at 18 months, that is, once the C1q expression significantly increased. These observations suggest that the permanent activation of C1q is partially related to the aging phenotypes caused by the loss of PGRN [120]. Suppressing complementary activation through deleting the C1qa gene may thus significantly enhance the survival rate, and decline neuro-degenerative phenotypes in the PGRN KO mice [119]. Drug targeting, using the C1 esterase inhibitor (C1-INH) reduced cardiac hypertrophy in an animal model. Moreover, C1-INH attenuates the β-catenin activation in the aged KO mice, which matches the results, stating the fact that C1q plays a central role in the regulation of the β-catenin pathways [120]. Besides, C1-INH ameliorates angiotensin II-induced arterial remodeling [131]. Therefore, this research line needs more investigations to move toward clinical translation.

## PGRN as lysosomal regulatiors of inflammation

Lysosomes are a single ubiquitous membrane-enclosed intracellular organelle with an acidic pH, present in all eukaryotic cells, that contains a large number of hydrolytic enzymes such as proteases, nucleases, and phosphatases that are able to degrade extra- and intracellular components [132]. Lysosomes are thus recognized as the key organelles for cellular clearance and are involved in many cellular processes to maintain cellular homeostasis [133]. Although the initial characterization of PGRN function was primarily focused on its role in extracellular signaling as a secreted protein, more recent studies have revealed the critical roles of PGRN in regulating lysosomal function, including proteolysis and lipid degradation, consistent with its localization [134]. In the case of CVD, many studies have further demonstrated that abnormal autophagy, including autophagic flux, can have a wide variety of pathogenic actions in the pathogenesis of this disease [135]. Another important aspect to consider is the role played by lysosomes in the activation of the NOD-, LRR- and pyrin domain-containing protein 3 (NLRP3) inflammasome, which can result in the maturation and release of IL-1β, a cytokine with a fundamental role in establishing and driving the pathogenesis of atherosclerosis [136]. Autophagy inhibition and the associated lysosome dysfunction also induce the formation and activation of NLRP3 inflammasomes. Lysosomes play a decisive role in cytokine release during atherosclerosis progression. Particularly, secretory lysosomes facilitate the release and degradation of cytokines that require non-conventional secretion [137]. The deficiency of the lysosomal-associated membrane protein-2 (LAMP-2) gene, which encodes for a lysosomal membrane protein on chromosome X, can accordingly cause Danon disease, which often leads to cardiomyopathy and heart failure. In human cardiomyocytes, autophagosome-lysosomal fusion also requires LAMP-2 isoform B [138]. Although PGRN has been reported to play signaling roles as a secreted protein, growing evidence indicates that it is a key regulator of lysosomal degradative processes via cathepsin and glucocerebrosidase regulation, particularly in the brain [134]. PGRN has also been identified as a CatD chaperone, suggesting a role in lysosomal proteolysis, which stimulates CatD activity in cell-free assays due to physical, stabilizing interactions [139–141]. More recent studies have thus suggested that PGRN and partial-length, multi-GRN peptides bind to immature CatD, facilitating its conversion into mature types at acidic pH [142, 143]. PGRN may also be physiologically involved in lysosomal lipid catabolism [144]. Aside from the role of PGRN in lysosomal proteolysis and lipolysis, there are clues that it may play broader roles in the endolysosomal compartment [145]. Sortilin 1 (SORT1) is also a multiligand type I transmembrane protein belonging to the vacuolar protein sorting family that is located both on the cell surface and in endolysosomal compartments [146]. The function of the SORT1 receptor is mainly related to the transport of proteins of various types from the cell surface to the intracellular compartments, such as lysosomes and endosomes, via the Golgi apparatus in neuronal and non-neuronal cells [146, 147]. In this case, after the binding of PGRN to SORT1, the entire resulting ligand-receptor complex undergoes endocytosis from the extracellular space, which is associated with the further delivery of PGRN to the lysosome, which may also take place in a mechanism independent of SORT1 through interaction with prosaposin [148, 149]. It can also be assumed that the free C-terminal end of cleaved PGRN mediated the interaction with SORT1. The regulation occurring within this signal axis can in this case be seen as an endogenous mechanism of regulation of the extracellular level of PGRN and turnover at the level of endocytosis/exocytosis phenomena, which has been demonstrated both during in vitro and in vivo tests [148, 150].

## Potential role of PGRN in inflammation and SARS-CoV-2 (COVID-19)

It was observed that patients with COVID-19 were facing significant risks for 20 types of cardiovascular diseases, including myocardial infarction 1 year after infection [151]. Studies have further shown that cardiac complications can occur even in people with a mild form of the disease. However, cardiac complications had a higher frequency in a group hospitalized in the intensive care unit (ICU) due to severe disease forms [152]. Considering the widespread global outbreak of the disease, physicians wonder whether this pandemic causes cardiovascular complications. A study of over 500.000 patients with COVID-19 also revealed a 167% higher risk of thrombosis in the cases with SARS-COV-2 within 2 weeks of infection than in influenza [153]. The effect of SARS-COV-2 on the heart could be further related to angiotensin-converting enzyme 2, wherein the virus was employed for entering the cells. COVID-19 is thus considered a disorder with severe and systemic inflammation that leads to phenomena, such as acute respiratory distress syndrome (ARDS) and the failure of vital organs, including the heart [154, 155]. Studies have further demonstrated that recruiting neutrophils and monocytes in COVID-19 to various organs results in injuries and disorders. On the other hand, the destructive role of Th17 in developing this phenomenon has been established [156]. SARS-CoV-2 raises strong inflammatory responses including cytokine storm, initiating from the lungs and spreading to the heart, which induces viral myocarditis and elevated troponin levels in the bloodstream, and leads to fatal heart failure. Indeed, inflammation, particularly the multisystem inflammatory syndrome in children (MIS-C), has been currently established as the cause of heart failure. COVID-19 has been further reported to augment the risk of cardiac shock, arrhythmia, and sometimes sudden death in patients with CVDs [157]. Furthermore, drug interactions with the targeted therapies for COVID-19 may predispose the cases with underlying CVD to a higher risk of cardiomyopathy, arrhythmias and sudden death. Vascular inflammation and cardiac damage also occurs in 20-30% of hospitalized COVID-19 patients and account for 40% of deaths. Cytokine release syndrome (CRS) may further lead to elevated cytokine levels, and dysfunctions in the T lymphocyte along with lymphocytopenia at the early stages, and increase significantly at the later stages of COVID-19. This is typically associated with increased levels of CRP, cytokines such as IL-2 and IL-6, and cardiac natriuretic peptides [154]. These factors may all give rise to heart dysfunction or inflammation due to the growth in the atrial natriuretic factor and high serum ferritin levels, which may result in arrhythmia, myocardial dysfunction, heart failure, or stress cardiomyopathy. In some patients with COVID-19, the immune cells also cause inflammation by releasing cytokines and chemokines that can influence coagulation and clotting through multiple pathways. For example, the activation of the complement system after viral entry can lead to thrombosis [158]. Therefore, considering the similarity of the immunopathogenesis of heart failure following COVID-19 with other cases reviewed in this article, PGRN, as a regulator of inflammatory responses affecting the critical immunological events involved in the occurrence of damage caused by inflammation, can be proposed as a therapeutic and diagnostic target in the type of CVDs caused by COVID-19.

## Conclusion

The presence of immunological cells and mediators as well as their roles in maintaining proper cardiac functions have thus far been clarified. In this regard, dysregulated immune responses and uncontrolled inflammation are the key factors in developing various types of CVDs. As a regulatory and mainly anti-inflammatory factor whose role has been shown in many and autoimmune diseases, PGRN can be thus considered as a missing link in the chain of the events of CVD immunopathogenesis. PGRN in the heart can thus essentially contribute to the retention of homeostasis against aging, and the overload of blood pressure. The methods for enhancing PGRN expression accordingly show new therapeutic prospects for preventing cardiac dysfunction and hypertrophy. The PGRN deficiency further accelerates age-associated cardiac aging, hypertrophy, and dysfunction through C1q-induced β-catenin. The administration of PGRN accordingly protects against acute myocardial ischemia. It also improves cardiac function, possibly indicating the essential therapeutic and physiological function of PGRN in ischemic heart disease. However, there is a dire need for future clinical studies to identify the PGRN contribution to obesity-related ischemic heart disease. The PGRN therapy in a mouse model of acute myocardial ischemia-reperfusion injury had thus led to major improvements in cardiac function. PGRN also reverses the ischemia impact on cardiac function and reduces cellular damage. Another remarkable conclusion is the effectiveness of rPGRN in treating the mice with hyperhomocysteinemia. The recombinant PGRN administration also decelerates the development of diseases and consequently ameliorates the hyperhomocysteinemia-induced cardiac damage, suggesting the necessity of PGRN for cardioprotection as a novel treatment prospect to deal with hyperhomocysteinemia patients. The GRN also speeds up the calcification of VICs by activating NF-κB, Akt, as well as Smad1/5/8 pathways. In addition, PGRN may protect against myocardial ischemia-reperfusion injury by modulating the post-ischemic inflammatory response. The increased expression of PGRN following the MI induction is further related to its protective contributions against myocardial ischemia-reperfusion injury. Thus, the impact of PGRN under MI conditions must be understood. Finally, the dynamic changes in the PGRN localization and expression after MI bring the treatment potentials for myocardial ischemia-reperfusion injury.

## Data Availability

The datasets used and/or analyzed during the current study are available from the corresponding author on reasonable request.
